# *In Vitro* and* In Vivo* Anti-*Helicobacter* Activities of* Eryngium foetidum* (Apiaceae),* Bidens pilosa* (Asteraceae), and* Galinsoga ciliata* (Asteraceae) against* Helicobacter pylori*

**DOI:** 10.1155/2016/2171032

**Published:** 2016-08-18

**Authors:** Laure brigitte Kouitcheu Mabeku, Bertrand Eyoum Bille, Eveline Nguepi

**Affiliations:** ^1^Microbiology and Pharmacology Laboratory, Department of Biochemistry, Faculty of Science, University of Dschang, P.O. Box 67, Dschang, Cameroon; ^2^Gastroenterology Department, Laquintinie Hospital of Douala, P.O. Box 4035, Douala, Cameroon

## Abstract

This study was performed to evaluate the antimicrobial activities of extracts of* Bidens pilosa, Galinsoga ciliata,* and* Eryngium foetidum* against 6 clinical strains of* Helicobacter pylori in vitro* and* in vivo*. Broth microdilution method was used* in vitro*.* In vivo*, Swiss mice were inoculated with* H*.* pylori* and divided into 5 groups; the control group received the vehicle and the four others received 125, 250, and 500 mg/kg of methanol extract of* Eryngium foetidum* and ciprofloxacin (500 mg/kg) for 7 days, respectively.* Helicobacter pylori* colonization and number of colonies in gastric biopsies culture were assessed on days 1 and 7 after treatment. The lowest MIC value (64 *μ*g/mL) and the best spectrum of bactericidal effect (MBC/MIC = 1) were obtained with the methanol extract of* Eryngium foetidum*. The number of* H*.* pylori* infected animals was 17% (plant-extract) and 0% (ciprofloxacin) compared to 100% for the infected untreated group. Plant-extract (381.9 ± 239.5 CFU) and ciprofloxacin (248 ± 153.2 CFU) significantly reduced bacterial load in gastric mucosa compared to untreated, inoculated mice (14350 ± 690 CFU).* Conclusion*. The present data provided evidence that methanol extract of* Eryngium foetidum* could be a rich source of metabolites with antimicrobial activity to fight* Helicobacter pylori* infections.

## 1. Introduction

*Helicobacter pylori* is a Gram negative microaerophilic helical bacillus that affects the gastric mucosa and can be found attached to epithelial cells of the human stomach [1]. It is an etiologic agent of peptic ulcer disease, primary gastritis, gastric mucosa-associated lymphoid-tissue lymphoma, and gastric adenocarcinoma [2]. Approximately 20% of persons infected with* H*.* pylori* develop related gastroduodenal disorders during their lifetime [3]. Approximately 50% of persons are* Helicobacter pylori* positive around the world, with the developing countries having a prevalence of 80%–90% and industrialized countries like the United States having ≈35%–40% [3]. As the prevalence, the annual incidence of* H*.* pylori* infection is high in developing countries (≈4%–15%) compared with industrialized countries (0.5%) [4]. Low socioeconomic status, overcrowding, poor sanitation or hygiene, and living in a developing country are the risk factors of infection [2]. The recurrence of gastroduodenal disorders is substantially reduced by the eradication therapy of* H*.* pylori* infection. This eradication therapy entails two of the following antimicrobial agents; metronidazole, amoxicillin, tetracycline, or clarithromycin in association with a proton pump inhibitor or bismuth [5]. Quadruple regimens are used as a salvage therapy when triple therapy regimens failed [5]. The emergence of drugs resistance* H*.* pylori* strain is the most common causes of treatment failure [6]. The failure of 38% and 55% of cases, respectively, to therapy regimens containing metronidazole and clarithromycin has been documented, especially when these antimicrobial agents are used to treat infection with an organism resistant to one of the latter [7]. Consequently, there is the necessity for the development of new antimicrobial agents from natural sources for better chemotherapeutic applications [8]. Literature clearly shows the* in vitro* assessment of anti-*Helicobacter* properties of medicinal plant products [9–14]. However, because of short stomach transit time and poor acid stability, many compounds with* in vitro* anti-*Helicobacter* properties become ineffective* in vivo* [15–17]. Therefore, the* in vitro* therapeutic activity of plant products must be confirmed by an* in vivo* test. Even though no animal model of the* H*.* pylori* infection exactly matches the human disease, experimental models on some animals such as primates, domestic cats and dogs, gnotobiotic piglets, ferrets, Mongolian gerbils, and mice provide important insights into the* in vivo* evaluation of new antimicrobials.

*Eryngium foetidum* is an annual small plant (approximately 30 cm high) in the family Apiaceae, used as a medicinal herb and spice. It is used as health foods in view of its significant amount of calcium, iron, carotene, riboflavin, proteins, and vitamins A, B, and C content [18]. In traditional medicine, this plant is used in the treatment of various ailments such as fevers, chills, vomiting, burns, hypertension, headache, earache, stomachache, asthma, arthritis, snake bites, scorpion stings, diarrhea, malaria, and epilepsy [19, 20]. In addition, the aerial part of this plant is a rich source of essential oils which can be exploited in the cosmetic industry [20]. Eryngial (E-2-dodecenal) is the main constituent of volatile compounds of the plant [21]. Studies demonstrating the anthelmintic, anti-inflammatory, analgesic, anticonvulsant, anticlastogenic, anticarcinogenic, antidiabetic, and antibacterial activity of this plant have been reported [22–25].

Despite its popular uses to calm stomach pain, its activity against* H*.* pylori* which is a major cause of gastric ulcer has not been experimentally confirmed [26]. This study was therefore carried out to evaluate the* in vitro* and* in vivo* antibacterial activity of* Eryngium foetidum* on clinical strains of* H*.* pylori* in order to identify potential sources of materials for the synthesis of new drugs to fight* Helicobacter pylori* infections.

## 2. Materials and Methods

### 2.1. Bacterial Strains

The clinical microorganisms used here were obtained from the Gastroenterology Department of Laquintinie Hospital, Douala, Cameroon. These microorganisms were collected from gastric biopsies of patients complaining of upper gastrointestinal pain, hematemesis, or/and dyspepsia requiring gastroendoscopic examination. Six strains of* H*.* pylori* were freshly isolated and suspended in Brain Heart Infusion (BHI) broth supplemented with 5% horse serum and 20% glycerol and stored at −80°C until further use.

### 2.2. Animals

Swiss albino mice of 18–22 g weight obtained from Animal House, University of Dschang, Cameroon, were selected as experimental animals. These animals were fed with standard pellet diet and tap water.

### 2.3. Statement on Ethics Approval

Throughout the experiments, all animals received human care according to the criteria outlined in the internationally accepted principle guidelines of the European Union on Animal Care (CEE Council 86/609). Animal experiments were designated and approved by the Cameroon National Ethics Committee (Reg. number. FWA-IRB00001954).

### 2.4. Plant Material

The leaves of* Bidens pilosa* (Asteraceae) and* Galinsoga ciliata* (Asteraceae) were collected in Dschang (west region of Cameroon) and leaves of* Eryngium foetidum* (Apiaceaes) in Kumba (southwest region of Cameroon). Identification of the plant was done at the National Herbarium Yaoundé (voucher specimen numbers 42254/HNC, 57409/HNC, and 42131 HNC, resp., in the listed order. HNC: Cameroon National Herbarium). The plant materials were then air-dried at room temperature and ground into a fine powder.

### 2.5. Chemicals and Culture Media

Culture media (Columbia Agar, Brain Heart Infusion (BHI), lacked horse blood, Horse Serum, Vitox Supplement) and Campy*Gen* gas pack were obtained from Oxoid, Basingstoke, England. Doxycycline (doxycycline 200 mg, Combitic Global Caplet, India), metronidazole (metronidazole Tablets BP 500 mg, Strides Arcolab, India), and ciprofloxacin (zoflox, ciprofloxacin 750 mg, Odypharm) were used as reference antibiotics for the determination of the MIC and MBC and for the* in vivo* test were obtained from local pharmacy. P-Iodonitrotetrazolium chloride (INT, Sigma-Aldrich) was used to indicate microbial growth [27].

### 2.6. Extraction

Dried powdered plant material was weighed (250 g) and soaked in 3.5 L of ethyl acetate (EA) or methanol (MeOH) for 72 hours. The plant mixture was then filtered and concentrated under reduced pressure using a rotary evaporator. The extract was further concentrated by allowing it to stand overnight in an oven at 30°C.

### 2.7. *In Vitro* Study

#### 2.7.1. Antimicrobial Assay: Determination of MIC and MBC

The clinical strains (*H*.* pylori α1*,* H. pylori α2, H*.* pylori α3, H*.* pylori α4, H*.* pylori α5,* and* H*.* pylori α6*), identified using Gram staining and enzymatic properties (catalase, oxidase, and urease), were employed to evaluate the* in vitro* anti-*Helicobacter* activity of the plant-extracts. Methanol and ethyl acetate extracts of* Bidens pilosa*,* Galinsoga ciliata,* and* Eryngium foetidum* were used for the determination of MICs by the INT broth microdilution method [27] using 96-well plates. Twofold dilutions of each extract were prepared in the test wells in BHI broth supplemented with 5% horse serum (BHI-serum). The final extract concentrations ranged from 0.002 to 1.024 mg/mL. One hundred microliters of inoculums prepared from 48 h colonies on supplemented Columbia Agar (Columbia Agar + 5% (v/v) lacked horse blood and 1% (v/v) Vitox) at McFarland turbidity standard 3 was added to 100 *μ*L of the extract-containing culture medium. Control wells were prepared with culture medium and bacterial suspension and broth, only respectively. Ciprofloxacin, doxycycline, and metronidazole at concentration ranges of 0.002 to 0.128 mg/mL were used as positive control. The plates were covered with a sterile plate sealer; the contents of the wells were mixed with a shaker and incubated for 3 days at 37°C under microaerophilic conditions. After incubation, 40 *μ*L of 0.2 mg/mL INT was added per well and incubated at 37°C for 30 min. Living bacteria reduced the yellow dye to pink. The sample concentration that prevented the color change of the medium and exhibited complete inhibition of microbial growth is known as the MIC. The MBC was determined by adding 50 *μ*L aliquots of the preparations which prevented the color change of the medium after incubation during MIC assays, to 150 *μ*L of BHI-serum. These preparations were incubated at 37°C for 72 h under microaerophilic conditions. The lowest concentration of extract, which did not produce a color change after addition of INT, was considered as the MBC. Each MIC and each MBC were determined in triplicate and the mean values are recorded.

#### 2.7.2. Preliminary Phytochemical Analysis

The qualitative phytochemical analysis of* Eryngium foetidum* methanol extract was performed following the methods of Parekh and Chanda [28] to determine the presence of alkaloids (Mayer, Wagner, and Dragendorff test), flavonoids (alkaline reagent, Shinoda), phenolics (lead acetate, alkaline reagent test), triterpenes and steroids (Liebermann-Burchard Test), saponins (foam test), tannins (gelatine), anthraquinones (ether-chloroform 1 : 1 v/v, NaOH 10% w/v), and anthocyanins (1% HCl, heating). The results were qualitatively expressed in term of positive (+) and negative (−) signs.

### 2.8. *In Vivo* Anti-*Helicobacter pylori* Assessment

#### 2.8.1. Inoculation of Experimental Animals

Methanol extract of* Eryngium foetidum,* the most active plant-extract according to the MIC value obtained, was chosen for further* in vivo* anti-*Helicobacter* assessment. For this purpose, seventy-two mice were allowed to acclimatize for one week before initiation of experiment. After the acclimatization period, the animals were fasted for 12 h and sixty of them were infected with 0.2 mL of 10^8^ CFU/mL of* H*.* pyloriα*2 suspension, four times in one week with a 24 h interval between each inoculation. The inoculation day was considered as day 0 and subsequent days as day 1, day 2, and day 3 up to day 21. A group of uninfected mice serving as a normal group received sterile solution of the vehicle (200 *μ*L of 0.25% Tween 80).

#### 2.8.2. Distribution of Animals

Inoculated mice were distributed into five groups of 12 animals and allowed to rest for 24 h after the last inoculation. Group I, a negative control group, received sterile solution of the vehicle; group II was treated with ciprofloxacin (500 mg/kg) and served as positive control; test groups III to V were treated, respectively, with 125, 250 and 500 mg/kg of* Eryngium foetidum* extract, once daily for seven consecutive days. The mice were allowed to fast for 12 h after the last day of treatment and six from each group were sacrificed with ethyl ether. The rest of animal were sacrificed after a posttreatment period of seven days. The stomach of each animal was removed and opened through the longer curvature with sterile surgical instruments. The stomach and duodena, covering all subtypes of mucosa, were used for culturing* H*.* pylori*.

#### 2.8.3. Culture and Bacterial Load

Stomach biopsies were scraped off and homogenized with 0.5 mL of PBS in a 1.5 mL Eppendorf tube. The homogenized sample was serially diluted 10-fold. Each 0.1 mL of homogenate was plated on supplemented Columbia Agar and incubated at 37°C for 2 to 3 days under microaerophilic conditions [29].* H*.* pylori* was identified through macroscopic characteristics (small translucent colonies), microscopic characteristics and Gram staining (Gram negative curved rods), and enzymatic properties (urease, catalase, and oxidase positive). The bacterial load was estimated by colony count and expressed as log_10_ CFU per milliliter of homogenate.

### 2.9. Statistical Analysis

The results were expressed as means ± standard deviation. Before analysis, bacterial densities were expressed in log_10_. The analysis of variance followed by the paired Student's *t*-test was used for the statistical evaluation of data. The differences between groups were considered significant at *p* < 0.05.

## 3. Results

### 3.1. MIC and MBC Determination of Plant-Preparations and Antibiotics

Plant species showed different anti-*Helicobacter* activity with each other with MIC values ranging from 64 to >1024 *μ*g/mL. The methanol extract of* E*.* foetidum* shows the best activity for MIC values between 64 and 512 *μ*g/mL against 3/6 (50%) evaluated strains. Methanol extract from* E*.* foetidum* also displayed the best spectrum of bactericidal effect with a ratio of MBC/MIC 1 obtained on two tested* H*.* pylori* strains. MIC values from 128 to 512 *μ*g/mL were also recorded with* E*.* foetidum* (AE),* Bidens pilosa* (MeOH),* Bidens pilosa* (AE),* Galinsoga ciliate* (MeOH), and* Galinsoga ciliata* (AE) extracts, respectively, against 2/6 (33.33%), 4/6 (66.66%), 1/6 (16.66%), 4/6 (66.66%), and 4/6 (66.66%) of the evaluated bacteria. The best MIC value (64 *μ*g/mL) was obtained with the methanol extract from* E*.* foetidum* against* H*.* pylori α1* and* H*.* pylori α2*.

### 3.2. Phytochemical Screening

According to Table 2, alkaloids, phenols, flavonoids, anthraquinones, and sterol are the chemical compounds present in the methanol extract of* E*.* foetidum*.

### 3.3. Culture

After the cessation of treatment, the number of* H*.* pylori* positive animals in treated groups (plant-extract and ciprofloxacin) was lower than the number recorded in the untreated infected group (control group) (Table 3). None of the animals treated with the highest dose of plant-extract (500 mg/kg) or ciprofloxacin was* H*.* pylori* positive. However, only 83.33% of animals treated with 125 and 250 mg/kg of the plant-extract were* H*.* pylori* negative. There were no differences among the groups treated with three doses of plant-extract and ciprofloxacin at the second posttreatment period of time. Almost all the infected treated animals were* H*.* pylori* negative at the second posttreatment period, indicating the lasting action of the plant-extract or ciprofloxacin.

### 3.4. Bacterial Load

**Figure 1:**
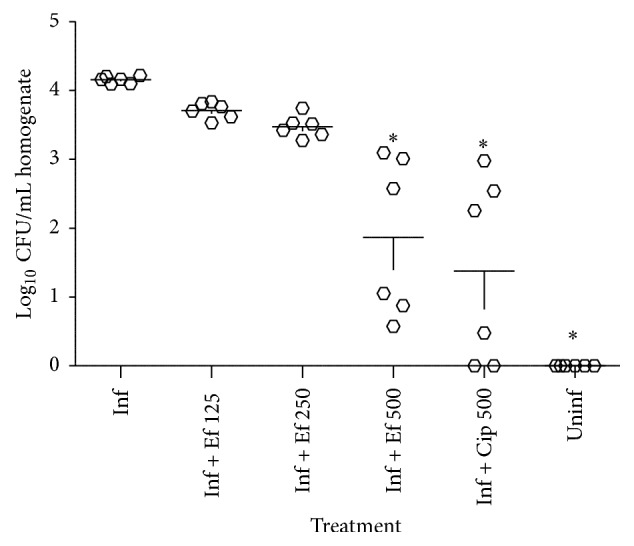
Effects of different treatment on the recovery of* H*.* pylori* from infected mice. Each circle represents the bacterial count for one animal. Inf: infected + vehicle; Inf + Ef 125: infected +* E*.* foetidum pinnatum* 125 mg/kg; Inf + Ef 250: infected +* E*.* foetidum *250 mg/kg; Inf + Ef 500: infected +* E*.* foetidum *500 mg/kg; Inf + Cp 500: infected + ciprofloxacin 500 mg/kg; Uninf: normal group. Data column of the different treatment with superscript *∗* are significantly different compared with infected control group (^*∗*^ *p* < 0.05).

Infected mice showed stable* H*.* pylori* colonization of gastric mucosa as shown in the number of bacterial counts (Figure 1). The bacterial load of gastric mucosa in infected mice was significantly reduced following the administration of 500 mg/kg of* E*.* foetidum* (381.9 ± 239.5 CFU) and ciprofloxacin (248 ± 153.2 CFU) compared to that recorded with untreated infected mice (14350 ± 690 CFU).

## 4. Discussion

In this study, the antimicrobial activities of the ethyl acetate and methanol extract of three medicinal Cameroonian plants were evaluated against six* H*.* pylori* strains.* In vitro*, the tested plants displayed selective antibacterial activities and this activity was different within the same plant species from one extraction solvent to another. In fact, the type of solvent used largely affects the effectiveness of the extracts. The differences observed in the antibacterial activities of the plants species could be due to the differences in their chemical composition and in the mechanism of action of their bioactive constituents [30]. It is known that the antibacterial activity of a plant-extract is considered significant when MIC values are less than 100 *μ*g/mL, moderate when being between 100 and 625 *μ*g/mL, and weak when being greater than 625 *μ*g/mL [31]. Thus, the MIC value of 64 *μ*g/mL obtained with* E*.* foetidum *methanol extract against* H*.* pylori α1* and* H*.* pylori α2* can be considered significant. The active principles of the extract of this plant should be assignable to different secondary metabolites belonging to a wide variety of compound classes. The preliminary phytochemical analysis of the tested plant-extract revealed the presence of alkaloids, phenols, flavonoids, anthraquinones, and steroid (Table 2). The result of the antimicrobial activity of* E*.* foetidum *obtained herein correlates with that of Lingaraju et al. [22] showing that the ethyl acetate extracts of this plant have antimicrobial activity against* Bacillus subtilis*,* Staphylococcus aureus*,* Escherichia coli*,* Pseudomonas aeruginosa,* and fungus* Candida albicans* using agar well diffusion method. The activity of the methanol extract of* E*.* foetidum *against* H*.* pylori α1* and* H*.* pylori α2* was similar to that of doxycycline, a standard drug against* H*.* pylori α1* (Table 1). This is quite remarkable particularly because the standard antibiotics are in the purified form whereas the extracts are mixtures of both pharmacological and nonpharmacological substances. Anti-*Helicobacter* activity of the methanol extract of* E*.* foetidum *was better than that of metronidazole, another antibiotic used in the treatment of* H*.* pylori* infection. Metronidazole-containing regimens have recently been shown to have limited effectiveness because of the increasing prevalence of resistance to this drug [32]. Its prevalence varies from 10 to 90% in different countries [33]. Studies of Ndip et al. [34] and Tanih et al. [32] carried out, respectively, in Cameroon and South Africa, documented a very high resistance rate of 95.5% to metronidazole.

Since the* in vitro* anti-*Helicobacter* properties of a compound can become ineffective* in vivo,* the most active plant-extract* in vitro* was also evaluated* in vivo* on the mouse model. As indicated by the confirmation tests for the presence of* H*.* pylori* and bacterial load, both methanol extract of* E*.* foetidum *(500 mg/kg) and ciprofloxacin significantly reduced* H*.* pylori* colonization of gastric mucosa in infected mice as compared to infected untreated mice. Thus, our data showed that the anti-*Helicobacter* activity of the methanol extract of* E*.* foetidum *was effective* in vitro* and* in vivo*. The activity of the plant-extract demonstrated herein is very important when considering that the tested plant-extracts are from edible plant parts and also when considering the medical importance of the tested bacteria. In fact,* H*.* pylori* is a virulent pathogen as evidenced by its low infective dose and high prevalence in human populations and the principal cause of type B gastritis, peptic ulcer disease, gastric adenocarcinoma, and MALT-lymphoma [35]. It has also been classified as a Class I carcinogen by the World Health Organization [35]. Furthermore, the continuous emergence of drugs resistant to* H*.* pylori* drastically reduced the limited range of antibiotics that have efficacy in the treatment of this infection leading globally to an increase in the frequency of therapeutic failure [36].

In conclusion, the present data provided evidence that methanol extract of* E*.* foetidum* could be a rich source of metabolites with antimicrobial activity to fight* Helicobacter pylori* infections. Further investigations on purification and structure elucidation of the compounds are in progress.

## Figures and Tables

**Table 1:** MIC/MBC of crude plant-extracts and antibiotics (*µ*g/mL).

Crude extracts/antibiotics	*H. pylori stains*					
	*H. pylori α1*	*H. pylori α2*	*H. pylori α3*	*H. pylori α4*	*H. pylori α5*	*H. pylori α6*
*E. foetidum *(MeOH)	64/64	64/64	512/512	>1024	>1024	>1024
*E. foetidum *(AE)	256/256	512/512	>1024	>1024	>1024	>1024
*Bidens pilosa (*MeOH)	1024/1024	512/1024	>1024	256/1024	512/1024	256/512
*Bidens pilosa (*AE)	1024/1024	>1024	>1024	256/512	>1024	1024/1024
*Galinsoga ciliata (*MeOH)	512/1024	256/512	>1024	>1024	128/512	256/512
*Galinsoga ciliata (*AE)	256/512	>1024	256/512	256/512	>1024	128/512
Doxycycline	64/64	32/32	32/32	32/32	4/16	32/32
Ciprofloxacin	8/8	32/32	2/2	<2	16/16	8/8
Metronidazole	>128	>128	>128	>128	>128	>128

All the values given in the table are means of three determinations. MeOH: methanol; AE: ethyl acetate.

**Table 2:** Phytochemical screening of methanol extract of *E. foetidum*.

Compounds	Methanol extract of *E. foetidum*
Alkaloids	+
Flavonoids	+
Phenolics	+
Saponins	−
Tannins	−
Triterpenes	−
Anthraquinones	+
Anthocyanins	−
Steroid	+

+: present; −: absent.

**Table 3:** Effects of different doses of *E. foetidum *and ciprofloxacin on *H. pylori* gastric colonization of infected and normal mice.

Treatment group (mg/kg)	Tests	1 day after cessation of treatment							7 days after cessation of treatment						
		Animals number							Animals number						
		1	2	3	4	5	6	Negative (%)	1	2	3	4	5	6	Negative (%)
Normal	Urease	−	−	−	−	−	−	100	−	−	−	−	−	−	100
	Catalase	−	−	−	−	−	−	100	−	−	−	−	−	−	100
	Oxidase	−	−	−	−	−	−	100	−	−	−	−	−	−	100
	Gram st	−	−	−	−	−	−	100	−	−	−	−	−	−	100

*H. pylori* + vehicle	Urease	+	+	+	+	+	+	0	+	+	+	+	+	+	0
	Catalase	+	+	+	+	+	+	0	+	+	+	+	+	+	0
	Oxidase	+	+	+	+	+	+	0	+	+	+	+	+	+	0
	Gram st	+	+	+	+	+	+	0	+	+	+	+	+	+	0

*H. pylori *+ *E. foetidum*, 125	Urease	−	−	−	+	+	+	50	−	−	+	+	−	−	67
	Catalase	−	−	−	+	−	−	83	−	−	−	−	−	−	100
	Oxidase	−	−	−	−	−	−	100	−	−	−	−	−	−	100
	Gram st	−	−	−	+	+	−	67	−	−	−	+	−	−	83

*H. pylori *+ *E. foetidum*, 250	Urease	−	+	+	−	−	−	67	−	−	−	−	−	+	83
	Catalase	−	−	−	−	−	−	100	−	−	−	−	−	−	100
	Oxidase	−	−	−	−	−	−	100	−	−	−	−	−	−	100
	Gram st	−	+	−	−	−	−	83	−	−	−	−	−	−	100

*H. pylori *+ *E. foetidum*, 500	Urease	−	−	−	−	−	+	83	−	−	−	−	−	−	100
	Catalase	−	−	−	−	−	+	83	−	−	−	−	−	−	100
	Oxidase	−	−	−	−	−	−	100	−	−	−	−	−	−	100
	Gram st	−	−	−	−	−	+	83	−	−	−	−	−	−	100

*H. pylori *+ ciprofloxacin, 500	Urease	−	−	−	−	−	−	100	−	−	−	−	−	−	100
	Catalase	−	−	−	−	+	−	83	−	−	−	−	−	−	100
	Oxidase	−	−	−	−	−	−	100	−	−	−	−	−	−	100
	Gram st	−	−	−	+	−	−	83	−	−	−	−	−	−	100

All the percentages given in the table are means of the six animals (*n* = 6) that tested negative per group. (+): positive test; (−): negative test.
